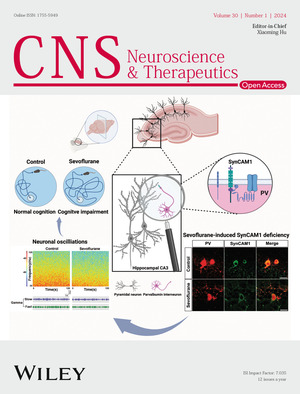# Front Cover

**DOI:** 10.1111/cns.14621

**Published:** 2024-01-29

## Abstract

The cover image is based on the Original Article *SynCAM1 deficiency in the hippocampal parvalbumin interneurons contributes to sevoflurane‐induced cognitive impairment in neonatal* by Ming‐ming Zhao et al., https://doi.org/10.1111/cns.14554.